# Evolution of Globus Pallidus Targeting for Parkinson's and Dystonia Deep Brain Stimulation: A 15-Year Experience

**DOI:** 10.3389/fneur.2021.679918

**Published:** 2021-08-12

**Authors:** Vanessa M. Holanda, Robert Stephen Eisinger, Leonardo Almeida, Takashi Tsuboi, Huimin Wang, Michael S. Okun, Wissam Deeb, Addie Patterson, Aparna Wagle Shukla, Janine Lobo Lopes, Kelly Douglas Foote

**Affiliations:** ^1^Department of Neurosurgery, Center of Neurology and Neurosurgery Associates (NeuroCENNA), Beneficencia Portuguesa of São Paulo Hospital, São Paulo, Brazil; ^2^Department of Neurology, Norman Fixel Institute for Neurological Diseases, University of Florida Health, Gainesville, FL, United States; ^3^Department of Neurology, Nagoya University, Nagoya, Japan; ^4^Department of Neurology, University of Massachusetts Medical School, Worcester, MA, United States

**Keywords:** deep brain stimulation, globus pallidus, targeting, dystonia, Parkinson's disease

## Abstract

**Objective:** The aim of this study is to evaluate the evolution of GPi DBS targeting.

**Methods:** This retrospective, single-center study included patients implanted with GPi DBS leads for dystonia or PD during the years 2004 to 2018 at the University of Florida Fixel Institute for Neurological Diseases. Each patient underwent a high-resolution targeting study on the day prior to the surgery, which was fused with a high resolution CT scan that was acquired on the day of the procedure. Intraoperative target location was selected using a digitized 3D Schaltenbrand-Bailey atlas. All patients underwent a high-resolution head CT scan without contrast approximately one month after lead implantation and accurate measurement of neuroanatomical lead position was acquired after fusion of pre-operative and post-operative image studies.

**Results:** We analyzed 253 PD patients with 352 leads and 80 dystonia patients with 141 leads. During 15 years of follow-up, lead locations in the PD group migrated more laterally (β = 0.09, *p* < 0.0001), posteriorly [slope (β) = 0.04, *p* < 0.05], and dorsally (β = 0.07, *p* < 0.001), whereas leads in the dystonia group did not significantly change position aside from a trend in the dorsal direction (β = 0.06, *p* = 0.053).

**Conclusion:** The evolving target likely results from multiple factors including improvements in targeting techniques and clinical feedback intraoperatively and post-operatively. Our demonstrates the potential importance of a systematic post-operative DBS lead measurement protocol to ensure quality control and to inform and optimize DBS programming.

## Introduction

Deep brain stimulation (DBS) is a surgical therapy that uses a neurostimulator and one or multiple brain leads to modulate specific neural circuits (1). Modulation of the globus pallidus internus (GPi) is a well-established and highly effective therapeutic option for appropriately selected patients with Parkinson's disease (PD) and for patients with severe dystonia that is refractory to optimized medical therapy (2, 3). GPI DBS has the potential to improve both hypokinetic and hyperkinetic disorders (4). Although the antiparkinsonian effects of GPi DBS are well recognized, there remains debate about the underlying therapeutic mechanism (5) and the ideal position for lead implantation within the target. The posterolateral, somatosensory region of the GPi has been one of the most consistently utilized areas and has shown robust benefits across studies (3, 6, 7).

Despite established efficacy and safety, the clinical response to DBS may at times be variable among patients and dependent on a variety of factors including patient selection, appropriate target selection, and adequate surgical planning. Okun et al. (2005) evaluated causes of DBS failures in patients referred to tertiary movement disorders centers (8). Nearly half of the patients had sub optimally placed DBS electrodes, and 17% had leads that were not programmable. Half of the cohort required lead revision (8). Although several factors may influence lead placement, appropriate surgical planning is critical to the process.

In both PD and dystonia, accurate DBS targeting is a primary determinant of outcomes (9) and the evolution of improved direct targeting techniques over the last two decades has provided more effective stimulation with fewer side effects. The aim of this study is to evaluate the evolution of GPi DBS targeting over time through analysis of a large, single-center cohort.

## Methods

This is an IRB-approved, retrospective, single-center study including patients implanted with GPi DBS leads for dystonia or PD during the years 2004–2018 at the University of Florida Fixel Institute for Neurological Diseases. Informed consent was obtained. We confirm that we have read the Journal's position on issues involved in ethical publication and affirm that this work is consistent with those guidelines.

The standard-of-care surgical approach has been previously described by our group (10). On the day prior to the surgery, each patient underwent a high-resolution 3 Tesla MRI direct targeting study. The image study included a Gadolinium enhanced MPRAGE sequence and a Fast Gray Matter Acquisition T1 Inversion Recovery sequence (FGATIR), which was introduced in 2009. On the day of the procedure, a high resolution CT scan was acquired after the Cosman-Roberts-Wells (CRW) frame was attached to the patient's head. Both the MRI and the CT images were fused and intraoperative target location was selected using a digitized Schaltenbrand-Bailey atlas (11). The atlas was manually fitted to each patient's pre-operative imaging using three-dimensional (3D) scaling, shifting, and rotating. All patients underwent a high-resolution head CT scan without contrast approximately 1 month after implantation. This image study was fused to the pre-operative targeting MRI, which allowed accurate measurement of neuroanatomical lead position. Planned and measured lead positions were reverse normalized based on individual atlas-fits in order to place all leads within the Schaltenbrand-Bailey atlas coordinate space for comparison across patients. We analyzed the PD and dystonia cohorts separately. For analysis, we dropped the sign of the lateral position to combine data from the left and right hemispheres. Absolute error in all three dimensions and 3D Euclidean error was computed.

Data were filtered to remove outliers defined by a z-score greater than or less than three standard deviations away from the mean with respect to the final planned positions or measured positions in the lateral, A-P, and axial dimensions. Correlation analysis was used to identify associations between time (years) and variables of interest including lead positions and errors in final positions. The significance level was set at an alpha of 0.05. The statistical software R was used for all analyses.

## Results

### Patient Characteristics

A total of 253 PD patients with 352 leads and 80 dystonia patients with 141 leads were included after outlier analysis led to exclusion of 10 patients. Within the PD group, 152 patients had unilateral implants and 101 patients had bilateral implants. Within the dystonia group, 13 patients had unilateral implants and 67 patients had bilateral implants. Ages at the time of surgery were 63.61 +/− 9.12 (M +/− SD) years in the PD group and 44.40 +/− 21.94 years in the dystonia group.

### Evolution of the Lead Positioning and Error

**Figure 1:**
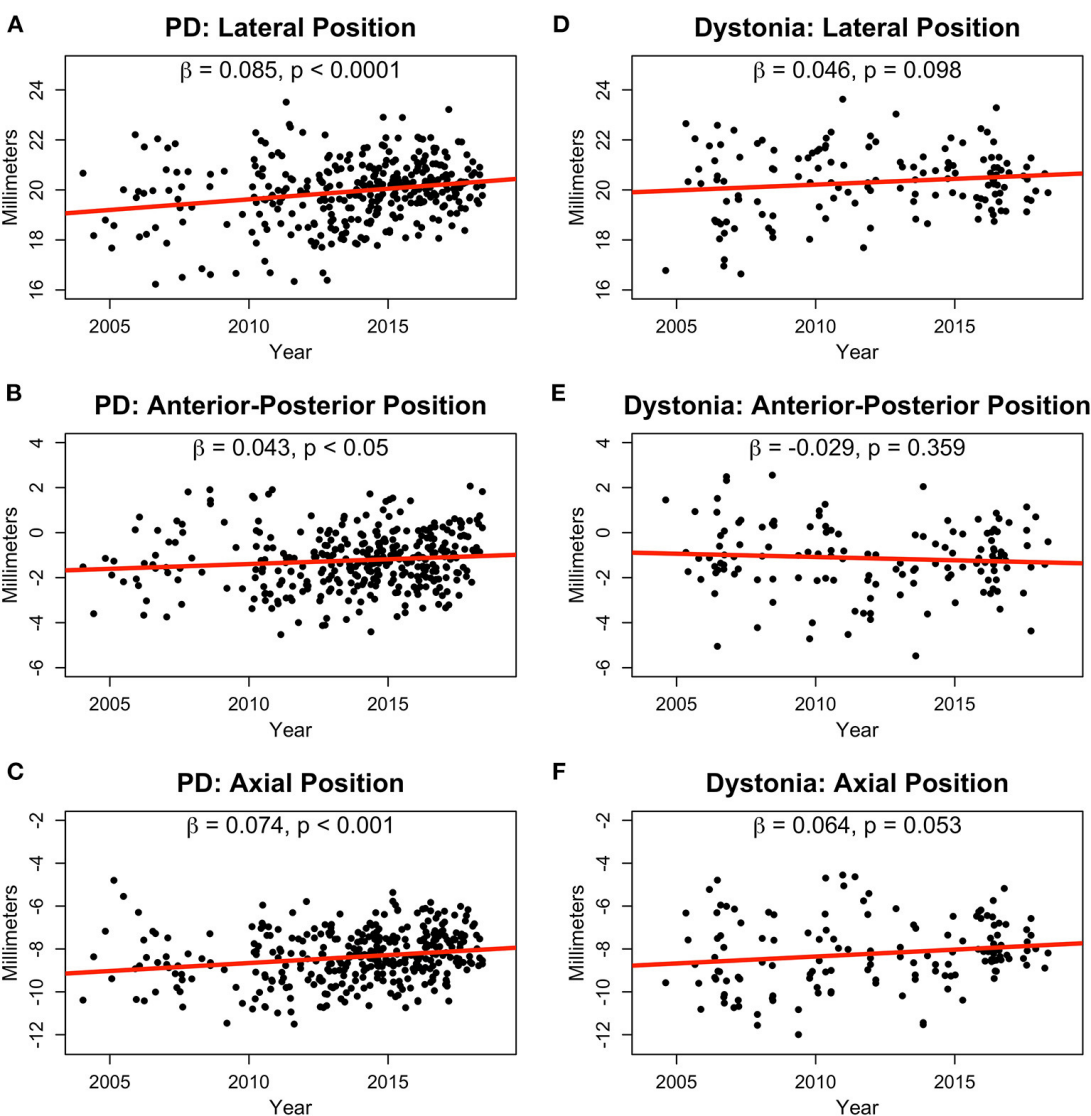
Normalized lead positions in millimeters for PD **(A–C)** and dystonia **(D–F)** patients in the lateral **(A,D)**, anterior-posterior **(B,E)**, and axial **(C,F)** dimensions shown from 2004 to 2018. Slopes (β) and *p*-values are shown for linear fits in all plots.

During 15 years of follow-up, lead locations in the PD group migrated more laterally (β = 0.09, *p* < 0.0001; Figure 1A), posteriorly [slope (β) = 0.04, *p* < 0.05; Figure 1B], and dorsally (β = 0.07, *p* < 0.001; Figure 1C), whereas leads in the dystonia group did not significantly change position aside from a trend in the dorsal direction (β = 0.06, *p* = 0.053; Figures 1D–F). Similar lateral (β = 0.11, *p* < 0.0001) and posterior (β = 0.09, *p* < 0.0001) shifts in the PD group were also found in the intraoperative planned coordinates whereas the axial shift (β = 0.02, *p* = 0.34) was not. However, the intraoperative planned coordinates for the lateral position in the dystonia group showed a small but significant shift more laterally with time (β = 0.05, *p* < 0.05).

**Figure 2:**
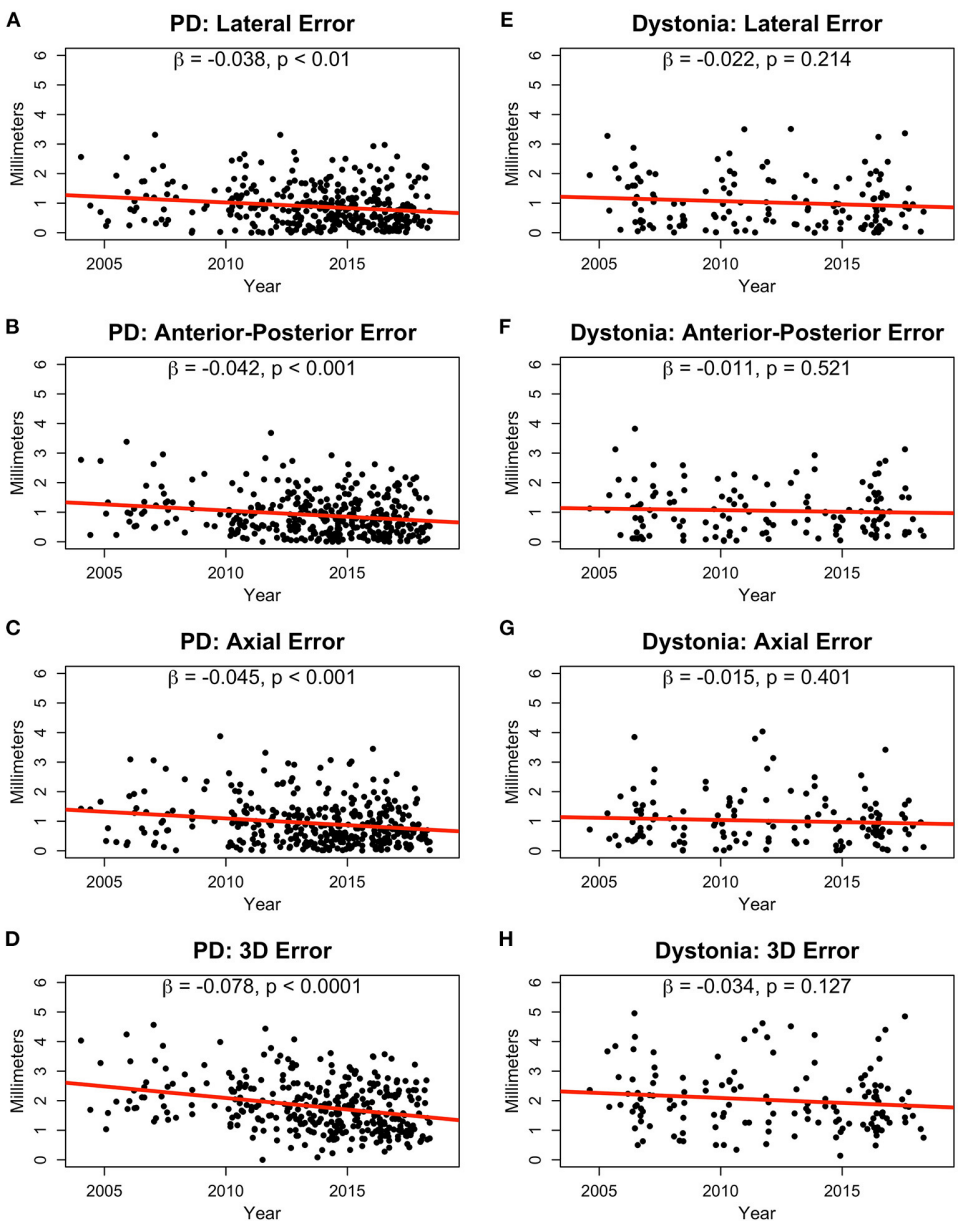
Absolute error in lead positions in millimeters for PD **(A–C)** and dystonia **(E–G)** patients in the lateral **(A,E)**, anterior-posterior **(B,F)**, and axial **(C,G)** dimensions shown from 2004 to 2018. 3D Euclidean distance in the PD **(D)** and dystonia groups **(H)** are also shown. Slopes (β) and *p*-values are provided for linear fits in all plots.

Absolute error significantly decreased over time in the PD group for the lateral (β = −0.04, *p* < 0.01; Figure 2A), A-P (β = −0.04, *p* < 0.001; Figure 2B), and axial (β = −0.05, *p* < 0.001; Figure 2C) directions leading to a reduction in overall 3D Euclidean error of about 0.08 mm per year (*p* < 0.0001; Figure 2D). In the dystonia group, error did not significantly reduce over time (Figures 2E–H). The average lead position from 2004–2006 to 2016–2018 for Parkinson's disease and dystonia in axial, coronal, and sagittal planes was shown in Supplementary Figure 1.

## Discussion

To our knowledge this is the first study evaluating long-term accuracy and evolution of GPi DBS targeting, though there are many studies evaluating the effectiveness of GPi DBS (12, 13). There is a paucity of data regarding errors in lead location measurement or of errors in surgical placement. It is well known in stereotactic surgery that the further away a target is from the midline, the higher the predicted error may be (14), especially if the targeting procedure is based only on indirect targeting relative to the mid-commissural point. Improvement of DBS targeting software and high resolution brain imaging [e.g., FGATIR, quantitative susceptibility mapping] has improved our ability to clearly identify and directly target neuroanatomical structures (15–17). The FGATIR sequence was introduced in 2009 and its combination of high resolution (1 x 1 x 1 mm voxel), 1 mm slice thickness and high contrast, enabled better visualization of the GPi borders, leading to a more precise location of the target (15). These improvements have likely contributed to the diminishing rate of GPi-DBS targeting errors during the more recent years of this cohort.

Evolution of the final lead placement may also be a result of technique refinement, including reasons beyond the advent of high-resolution MRI. For instance, clinical feedback from outpatient programming sessions may play a role as lateral placement decreases the likelihood of inadvertent internal capsule stimulation and widens the therapeutic window for DBS programming, an aspect particularly important in dystonia patients, who often require larger volumes of tissue activation to control their symptoms chronically (18). These patients are also more prone to stimulation-induced side effects. In addition, dystonia patients may require DBS earlier and require it for a longer time given the younger mean age at implantation. Dystonia patients commonly require higher settings and higher charge densities over time for chronic symptom control (19–22). This is the largest study to examine the evolution of GPi targeting over a period of 15 years, and it reinforces the potential importance of an interdisciplinary team and the clinical feedback directed from neurologists to neurosurgeons. We highlight that during these 15 years, there was consistency in our team in terms of senior neurosurgeon and senior neurology programmer. Clinical feedback relating lead locations to outcomes is particularly important for refining lead placements to maximize benefits while minimizing adverse effects of DBS (23). Surgical teams that do not receive such clinical feedback regarding their patients' post-operative lead locations relative to their clinical outcomes fail to exploit an important tool for DBS quality improvement and will likely have less optimal surgical outcomes.

There are some hypotheses for why the GPi targets moved through the years: (1) For the lateral move, it was mainly related to the neurological team feedback requiring more “room” to work. Over the years, due to the progression of the dystonia and Parkinson's disease, the DBS programming may require amplitude and pulse width increase to control the motor symptoms. (2) For the dorsal move, it could be related to the surgeon's cautious with deep vessels, which are commonly seen inferiorly to the GPi.

There are particular limitations of this analysis that should be mentioned. This is a single center study. As previously described by our group, microelectrode recording (MER) is part of our surgical approach. Thus, adjustments of the coordinates based on MER and intra operative stimulation can play an important role in the final location of the lead. The retrospective nature of the study can introduce bias related to record keeping, though this bias is partially offset by the large number of patients included. The different size of the heads between the patients can be a limitation; however, the planned and measured lead positions were reverse normalized to individual atlas-fits to place all leads within the Schaltenbrand-Bailey atlas coordinate space to enable more meaningful comparison across patients.

In summary, this is the first single tertiary center study to show the longitudinal evolution of targeting in GPi DBS. The evolving target likely results from multiple factors including improvements in targeting techniques and clinical feedback intraoperatively and post-operatively. Our results also demonstrate the potential importance of a systematic post-operative DBS lead measurement protocol to ensure quality control and to inform and optimize DBS programming. More precise targeting of the GPi with patient-specific modeling can potentially predict effectiveness by representing the volume of tissue activated. This strategy can aid in predicting optimal stimulation parameters, more efficiently maximizing therapeutic benefit and reducing adverse effects. Future studies incorporating a larger, multi-center dataset might be useful to corroborate the findings of this analysis and help to establish useful guidelines for improving electrode placement and global outcomes in movement disorders patients.

## Data Availability Statement

The raw data supporting the conclusions of this article will be made available by the authors, without undue reservation.

## Ethics Statement

The studies involving human participants were reviewed and approved by UF Institutional Review Board. The patients/participants provided their written informed consent to participate in this study.

## Author Contributions

VH: conception, organization and execution of research project, review and critique of statistical analysis, and writing the first draft of the manuscript. RE and TT: conception, organization and execution of research project, design, execution, review and critique of statistical analysis, and writing the first draft of the manuscript. LA: conception, organization and execution of research project, review and critique of statistical analysis, and review and critique of manuscript. HW: execution of research project, design, execution, review and critique of statistical analysis, and writing the first draft of the manuscript. MO and KF: conception of research project, review and critique of statistical analysis, and review and critique of manuscript. WD and AP: organization and execution of research project, review and critique of statistical analysis, and review and critique of manuscript. AW: review and critique of statistical analysis, and review and critique of manuscript. JL: review of manuscript. All authors contributed to the article and approved the submitted version.

## Conflict of Interest

The authors declare that the research was conducted in the absence of any commercial or financial relationships that could be construed as a potential conflict of interest.

## Publisher's Note

All claims expressed in this article are solely those of the authors and do not necessarily represent those of their affiliated organizations, or those of the publisher, the editors and the reviewers. Any product that may be evaluated in this article, or claim that may be made by its manufacturer, is not guaranteed or endorsed by the publisher.
