# Supplementary figures and images for: Evolution of Globus Pallidus Targeting for Parkinson's and Dystonia Deep Brain Stimulation: A 15-Year Experience
Source: Front Neurol. 2021 Aug 12;12:679918. doi: 10.3389/fneur.2021.679918 (PMC8387620; doi:10.3389/fneur.2021.679918)

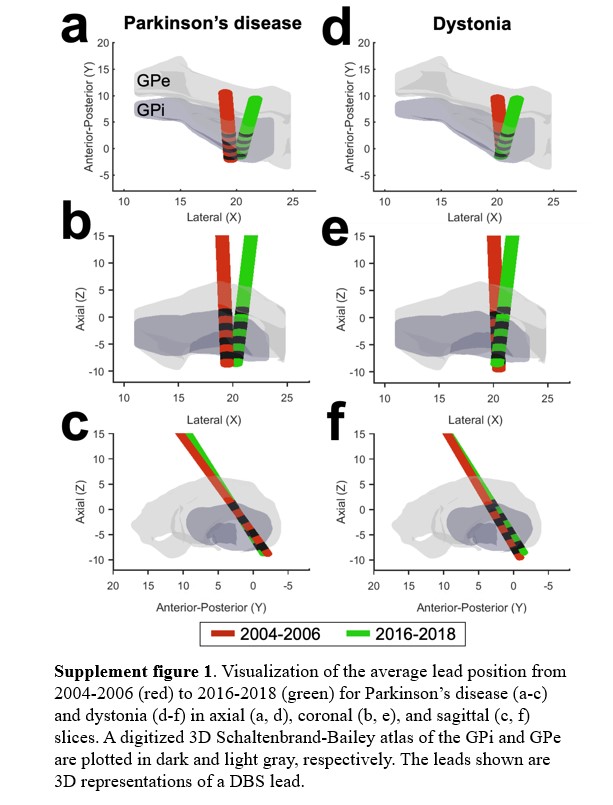

Supplement: Supplementary file 1 [file Image_1.JPEG]
